# Development and temporal evaluation of sex-specific models to predict 4-year atherosclerotic cardiovascular disease risk based on age and neighbourhood characteristics in South Limburg, the Netherlands

**DOI:** 10.1186/s41512-025-00198-4

**Published:** 2025-07-02

**Authors:** Anke Bruninx, Lianne Ippel, Rob Willems, Andre Dekker, Iñigo Bermejo

**Affiliations:** 1 Department of Radiation Oncology (Maastro), GROW Research Institute for Oncology and Reproduction, Maastricht University Medical Centre+, Maastricht, The Netherlands; 2 Statistics Netherlands, Heerlen, The Netherlands; 3 Data Science Institute, Hasselt University, Hasselt, Belgium

**Keywords:** Risk, Prognosis, Cardiovascular diseases, Environmental pollution, Socioeconomic factors

## Abstract

**Background:**

To improve screening for atherosclerotic cardiovascular disease (ASCVD), we aimed to develop and temporally evaluate sex-specific models to predict 4-year ASCVD risk in South Limburg based on age and neighbourhood characteristics concerning home address.

**Methods:**

We included 40- to 70-year-olds living in South Limburg on 1 January 2015 for model development, and 40- to 70-year-olds living in South Limburg on 1 January 2016 for model evaluation. We randomly sampled people selected in 1 year and in both years to create development and evaluation data sets. Follow-up of ASCVD and competing events (overall mortality excluding ASCVD) lasted until 31 December 2020. Candidate predictors were the individual’s age, the neighbourhood’s socio-economic status, and the neighbourhood’s particulate matter concentration. Using the evaluation data sets, we compared two model types, subdistribution and cause-specific hazard models, and eight model structures. Discrimination was assessed by the area under the receiver operating characteristic curve (AUROC). Calibration was assessed by calculating overall expected-observed ratios (E/O). For the final models, calibration plots were made additionally.

**Results:**

The development data sets consisted of 67,549 males (4-year cumulative ASCVD incidence: 3.08%) and 67,947 females (4-year cumulative ASCVD incidence: 1.50%). The evaluation data sets consisted of 66,068 males (4-year cumulative ASCVD incidence: 3.22%) and 66,231 females (4-year cumulative ASCVD incidence: 1.49%). For males, the AUROC of the final model equalled 0.6548. The E/O equalled 0.9466. For females, the AUROC equalled 0.6744. The E/O equalled 0.9838.

**Conclusions:**

The resulting model shows promise for further research. These models may be used for ASCVD screening in the future.

**Supplementary Information:**

The online version contains supplementary material available at 10.1186/s41512-025-00198-4.

## Background

Atherosclerotic cardiovascular disease (ASCVD) is one of the leading causes of mortality worldwide [1]. Precursors of ASCVD, such as hypertension and an adverse lipids profile, may go unnoticed, while, if known, these are possible to treat. Screening is therefore important. The European Society of Cardiology (ESC) states a cardiovascular risk assessment for a person without any known risk factors may be considered in males above 40 years and females above 50 years or after menopause [2]. However, this recommendation was issued with a low level of evidence. Furthermore, a systematic screening of this population would require intensive use of resources.

In the Netherlands, most people are registered in a primary care practice, where their name, sex, and home address are routinely collected. Using this information, ASCVD risk models can be developed based on age, sex, and neighbourhood characteristics associated with the person’s home address. The resulting estimate may be informative for a general practitioner when deciding to offer a clinical cardiovascular risk assessment to a patient without any other known risk factors.

Neighbourhood characteristics have been included in published multivariable cardiovascular risk models intended for clinical practice in different settings. The ASSIGN estimators for males and females are prediction models to assess cardiovascular disease risk for the Scottish population without previous cardiovascular illness [3]. These include the Scottish Index of Multiple Deprivation associated with the postal code of a person’s residence [4]. The Scottish Index of Multiple Deprivation is a summary measure based on indicators of income, employment, education, housing, health, and driving times to services, such as supermarkets and primary schools. Further, the QRISK models were developed to be used in primary care in the UK [5–7]. These models include the Townsend deprivation index associated with the person’s postal code [8]. The Townsend deprivation index combines employment, car-ownership, homeownership, and household overcrowding. Lastly, the PREDICT equations were developed based on primary care electronic health records in New Zealand [9]. These include quintiles of the New Zealand Index of Socioeconomic Deprivation associated with geographical units of 100 to 200 inhabitants [10]. The New Zealand Index of Socioeconomic Deprivation incorporates education, employment, income, homeownership, housing conditions (internet access, living space, humidity), and the proportion of single-parent families.

In these clinical prediction models, the included neighbourhood characteristics mostly focus on socio-economic status indicators. It may be worthwhile to consider environmental pollution in addition. The air pollutants particulate matter, ozone, and nitrogen dioxide, and the metals arsenic, cadmium, and lead have been researched repeatedly [11, 12]. Causal processes have been proposed linking particulate matter with a diameter < 2.5 µm, arsenic, cadmium, and lead to cardiovascular disease [12, 13]. Ozone and nitrogen dioxide have been associated with respiratory illness mostly [11].

In sum, we aim to develop and temporally evaluate multivariable prediction models for males and females to estimate 4-year ASCVD risk in South Limburg, the Netherlands. Candidate predictors will comprise age, socio-economic neighbourhood characteristics, and environmental pollution. These models may aid ASCVD screening in the future.

## Methods

### Sample selection

We created a development data set and an evaluation data set for males and females separately using data from Statistics Netherlands (the Dutch National Statistics Office). We selected all persons aged 40 to 70 (excl.) years and living in the healthcare region South Limburg on the 1 st of January 2015 [14–17]. Correspondingly, we selected all persons aged 40 to 70 (excl.) years and living in the healthcare region South Limburg on the 1 st of January 2016 [15–18]. Those uniquely selected in 2015 were randomly assigned with a probability of 50% to the development data set, and those uniquely selected in 2016 were randomly assigned with a probability of 50% to the evaluation data set. Those selected in both 2015 and 2016 were randomly assigned with a probability of 50% to either the development data set or the evaluation data set. We subsequently split the development data set and evaluation data set into males and females.

### Outcome

We predicted 4-year ASCVD risk, taking into account mortality due to other causes as a competing event. For each person, we noted the time from their inclusion date until an ASCVD event, mortality due to another cause, or censoring on the 31 st of December 2020. We defined ASCVD events corresponding to the Systematic Coronary Risk Evaluation 2 (SCORE2) models [19]. These models estimate ASCVD risk based on age, sex, smoking status, systolic blood pressure, total cholesterol, and high-density lipoprotein, and are endorsed by the ESC to use in clinical practice. The included International Statistical Classification of Diseases and Related Health Problems (10th Revision; ICD-10) codes indicating hospitalisation are I21, I22, I23, I61, I63, I64, I65, I66, I67 (excluding I67.1 and I67.5), I68 (excluding I68.2), and I69 [20]. The included ICD-10 codes indicating mortality due to ASCVD are: I10, I11, I12, I13, I15, I20, I21, I22, I23, I24, I25, I46, I47, I48, I49, I50, I51 (excluding I51.4), I52, I61, I63, I64, I65, I66, I67 (excluding I67.1 and I67.5), I68 (excluding I68.2), I69, I70, I71, I72, I73, and R96. The data source for hospitalisation due to ASCVD was the Dutch Hospital Data registry [21]. The data source for mortality was Statistics Netherlands [22, 23].

### Candidate predictors

We extracted a person’s age via data from Statistics Netherlands [17]. This variable was standardised (i.e., subtracting the mean, followed by dividing by the standard deviation) based on the development data sets before inclusion in the models.

In addition, we determined the neighbourhood associated with a person’s residence using the mapping of 2021 [15, 16, 24, 25]. We searched for routinely published, open data on socio-economic indicators, air pollution, arsenic, cadmium, and lead to link to the person’s neighbourhood.

Concerning socio-economic candidate predictors, we selected indicators of wealth and education published by Statistics Netherlands [26]. These data at the neighbourhood level are based on the households in the neighbourhood. Due to privacy concerns, data are not published when the neighbourhood consists of fewer than 100 households. Considering clinical implementation, missing values were imputed with the corresponding value calculated based on a larger geographical unit in between a neighbourhood and a municipality [27]. If this was missing as well, the value was imputed with the corresponding value calculated at the municipality level, which was never missing.

The wealth variable of the neighbourhood is based on the wealth of the households in the neighbourhood [26]. The wealth of a household is determined by the household’s disposable income, correcting for the household’s composition and the household’s assets. These are summed, and the percentile in which the household is situated in the Netherlands is calculated. The mean percentile of the households in the respective neighbourhood is the neighbourhood’s wealth indicator.

Regarding education, the level of education of the neighbourhood is based on the highest completed level of education of the household’s primary provider or their partner [26]. Three levels are used: a low level for primary or the first phase of secondary education, an intermediate level for subsequent secondary education, and a high level for tertiary education. The percentage of households with low, intermediate, and high education levels per neighbourhood is calculated. After imputation, we calculated the percentile per neighbourhood in the Netherlands.

Subsequently, we performed principal component analysis in the development data sets using the standardised wealth and education indicators and retained the first principal component as an indicator of socio-economic status [28].

Concerning air pollution, we linked particulate matter concentrations based on maps published by the Dutch National Institute for Public Health and the Environment [29]. We calculated the mean per neighbourhood of the modelled yearly average of 24 h of particulate matter with a diameter of < 2.5 µm per µg/m^3^. Subsequently, we calculated the percentile per neighbourhood in South Limburg. This variable was standardised based on the development data sets before inclusion in the models.

Lastly, we did not find suitable data regarding arsenic, cadmium, and lead.

The selected data were published yearly. If the inclusion date was the 1 st of January 2015, the published numbers of the year 2014 were used. Likewise, if the inclusion date was the 1 st of January 2016, the published numbers of the year 2015 were used.

### Modelling

Two model types were fitted: subdistribution and cause-specific hazard models [30, 31]. While subdistribution hazard models are most often used to estimate risk in a competing risk setting, the combined cumulative incidence may be higher than 100% [32]. This is not the case for cause-specific hazard models. Per model type, eight model structures were fitted. The first model structure included age as a linear term. The second structure included age and socio-economic status as linear terms. The third structure included age and particulate matter as linear terms. The fourth structure included age, socio-economic status, and particulate matter as linear terms. The fifth structure included age as a restricted cubic spline with 5 knots and socio-economic status as a linear term. The sixth structure included age as a restricted cubic spline with 5 knots and particulate matter as a linear term. The seventh structure included age as a restricted cubic spline with 5 knots, and socio-economic status and particulate matter as linear terms. The eighth and final structure included age, socio-economic status, as well as particulate matter as restricted cubic splines with 5 knots.

We assessed discrimination and calibration in the evaluation data sets. Discrimination was assessed by calculating the time-dependent area under the receiver operating characteristic (AUROC) curve for ASCVD [33]. This statistic indicates how well the model discriminates between those with an ASCVD event and those without. A value of 0.5 indicates the model does not discriminate better than random, while a value of 1 indicates a perfect discrimination. Calibration was assessed by calculating the expected-observed ratio, i.e. the ratio between the overall expected 4-year ASCVD risk and the observed 4-year ASCVD risk. The latter is the cumulative ASCVD incidence calculated via the Aalen–Johansen estimator, which takes into account competing risk [34]. Based on these results, we selected a final model for males and females, for which we present calibration plots comparing observed and predicted 4-year ASCVD risk per decile with locally estimated scatterplot smoothing using the evaluation data sets. Finally, we conducted a sensitivity analysis concerning the imputed values by assessing discrimination (AUROC) and calibration (expected-observed ratio) of the final models separately for the subset with complete cases and the subset with imputed values. Due to the low number of events in the subsets with imputed values, we did not draw calibration plots.

### Data availability

Data from Statistics Netherlands, the Dutch Hospital Data foundation, and the Dutch National Institute for Public Health and the Environment were combined. Open data are possible to access via the references. Part of the data is not publicly available.

### Software, code availability, and application

Data were analysed within a secure environment of Statistics Netherlands, using Python [35] with the pandas package [36] attached, and using R Statistical Software [37] with the following packages attached: cmprsk [38], ggplot2 [39], prodlim [40], raster [41], riskRegression [42], rms [43], sf [44], stars [45], and survival [46].

The code (excl. preprocessing of personal data) can be found on https://github.com/CARRIER-project/ASCVD-risk-models-neighbourhood. This includes the equations of the final models.

Furthermore, we made an application in R with the attachment of the packages bslib [47], ggplot2 [39], sf [44], and shiny [48]. A demo is available on https://abruninx.shinyapps.io/ASCVD-risk-models-neighbourhood. Here, postal codes are linked to neighbourhoods [49]. Please note the online application will be archived in the future. The code will remain available in the repository.

### Reporting

Reporting was guided by the Transparent Reporting of a Multivariable Prediction Model for Individual Prognosis or Diagnosis + Artificial Intelligence (TRIPOD + AI) guidelines [50]. Checklists are included in Supplementary Material 1.

## Results

### Description of the sample

Concerning males, the development data set included 67,549 persons, of which 2079 persons had an ASCVD event and 1611 passed away due to a different cause before 4 years of follow-up. The 4-year cumulative ASCVD incidence was 3.08%. The evaluation data set consisted of 66,068 persons, of which 2126 had an ASCVD event and 1465 passed away due to a different cause before 4 years of follow-up. The 4-year cumulative ASCVD incidence was 3.22%.

Concerning females, the development data set included 67,947 persons, of which 1018 persons had an ASCVD event and 1266 passed away due to a different cause before 4 years of follow-up. The 4-year cumulative ASCVD incidence was 1.50%. The evaluation data set consisted of 66,231 persons, of which 987 persons had an ASCVD event and 1149 passed away due to a different cause before 4 years of follow-up. The 4-year cumulative ASCVD incidence was 1.49%.

Table 1 provides for each data set the mean and standard deviation of age, wealth, education, and particulate matter concentrations. Concerning wealth and education, the imputed variables were used. The number of imputations ranged from 1449 to 1626.

**Table 1:** Description of the development and evaluation data sets

			Development	Evaluation
			Mean (SD)	Mean (SD)
Males	Age in years		56 (8)	55 (8)
	Wealth percentile		48 (11)	48 (11)
	Education percentile	Low	72 (24)	71 (25)
		Intermediate	44 (21)	45 (21)
		High	36 (27)	36 (27)
	PM percentile		60 (26)	60 (25)
Females	Age in years		56 (8)	55 (8)
	Wealth percentile		48 (11)	48 (11)
	Education percentile	Low	72 (25)	71 (25)
		Intermediate	44 (21)	44 (21)
		High	36 (27)	36 (27)
	PM percentile		60 (26)	60 (25)

*PM* particulate matter, *SD* standard deviation

### Development of the socio-economic status indicator

Table 2 presents Pearson’s correlation coefficients among wealth and education, separately for males and females. There are no substantial differences between the correlations for males and females. In addition to the expected correlations between the different levels of education, high correlations between wealth and low and high education levels are seen.

**Table 2:** Correlations among wealth and education in the development data sets for males and females

			Wealth percentile	Education percentile		
				Low	Intermediate	High
Males	Wealth percentile		1.00			
	Education percentile	Low	− 0.62	1.00		
		Intermediate	− 0.25	0.23	1.00	
		High	0.70	− 0.90	− 0.53	1.00
Females	Wealth percentile		1.00			
	Education percentile	Low	− 0.63	1.00		
		Intermediate	− 0.26	0.23	1.00	
		High	0.71	− 0.91	− 0.53	1.00

The wealth variable and education level variables were used to derive a principal component, i.e., socio-economic status. This principal component explained 90.88% of the variance in the male development data set and 91.15% in the female development data set. The loadings are presented in Table 3. Higher values of wealth and a high level of education lead to higher values of socio-economic status, while higher values on a low level of education and an intermediate level of education lead to lower values of socio-economic status, both for males and females.

**Table 3:** Loadings to construct the principal component for socio-economic status

			Loadings
Males	Wealth percentile		0.4819
	Education percentile	Low	− 0.5411
		Intermediate	− 0.3451
		High	0.5964
Females	Wealth percentile		0.4834
	Education percentile	Low	− 0.5415
		Intermediate	− 0.3448
		High	0.5951

### Model comparison

Table 4 presents the AUROCs and expected-observed ratios. Reviewing the AUROCs, the differences are rather minor. The highest value for males is observed for the model structure including age, socio-economic status, and particulate matter as restricted cubic splines, with equal values for the subdistribution hazard model and the cause-specific hazard model. The highest value for females is observed for the model including age, socio-economic status, and particulate matter as linear terms, with equal values for the subdistribution hazard model and the cause-specific hazard model.

**Table 4:** Area under the receiver operating characteristic (AUROC) curve and expected-observed ratio (E/O) values

		AUROC		E/O	
		SDH	CSC	SDH	CSC
Males	Age	0.6439	0.6439	0.9432	0.9430
	Age + SES	0.6523	0.6524	0.9431	0.9428
	Age + PM	0.6452	0.6452	0.9434	0.9431
	Age + SES + PM	0.6530	0.6530	0.9430	0.9428
	rcs(age, 5) + SES	0.6522	0.6522	0.9441	0.9438
	rcs(age, 5) + PM	0.6452	0.6452	0.9445	0.9442
	rcs(age, 5) + SES + PM	0.6529	0.6529	0.9440	0.9437
	rcs(age, 5) + rcs(SES, 5) + rcs(PM, 5)	0.6548	0.6548	0.9466	0.9463
Females	Age	0.6613	0.6613	0.9832	0.9829
	Age + SES	0.6738	0.6738	0.9841	0.9837
	Age + PM	0.6626	0.6627	0.9831	0.9828
	Age + SES + PM	0.6744	0.6744	0.9838	0.9834
	rcs(age, 5) + SES	0.6728	0.6728	0.9801	0.9796
	rcs(age, 5) + PM	0.6625	0.6625	0.9791	0.9787
	rcs(age, 5) + SES + PM	0.6733	0.6733	0.9798	0.9794
	rcs(age, 5) + rcs(SES, 5) + rcs(PM, 5)	0.6728	0.6728	0.9809	0.9804

*AUROC* area under the receiver operating characteristic, *CSC *cause-specific hazard model, *PM *particulate matter, *rcs *restricted cubic spline, *SDH *subdistribution hazard model, *SES *socio-economic status

Reviewing the expected-observed ratios, differences are rather minor as well. For males, the value closest to 1 is observed for the model including age, socio-economic status, and particulate matter as restricted cubic splines, both for the subdistribution hazard models and the cause-specific models. The value is slightly closer to 1 for the subdistribution hazard model. For females, the value closest to 1 is observed for the model including age and socio-economic status as linear terms, both for the subdistribution hazard models and the cause-specific models. The value is slightly closer to 1 for the subdistribution hazard model.

### Final models

As the final model for males, we selected the subdistribution hazard model including age, socio-economic status, and particulate matter as restricted cubic splines, given this model had the highest performance reviewing the AUROCs and expected–observed ratios.

As the final model for females, we selected the subdistribution hazard model including age, socio-economic status, and particulate matter as linear terms. This model had the highest AUROC and the second best expected–observed ratio.

**Fig. 1:**
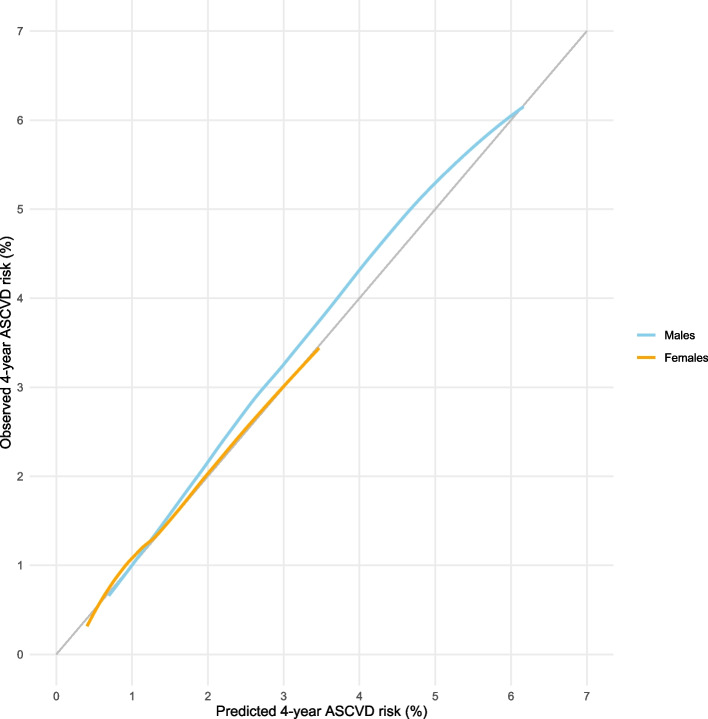
Calibration plot. ASCVD = atherosclerotic cardiovascular disease

Figure 1 presents the calibration plot for the final model for males and the final model for females. Regarding the final model for males, the predicted probabilities lower than 1.2% correspond quite well to the observed probabilities, while the predicted probabilities above 1.2% are slightly underestimated. Regarding the final model for females, the predicted probabilities lower than 0.6% are slightly overestimated, while the predicted probabilities in the range 0.6% to 1.3% are slightly underestimated. The probabilities above 1.3% correspond quite well to the observed probabilities. Calibration is deemed acceptable for both models.

Table 5 presents the results of the sensitivity analysis regarding the imputed values. Concerning the model for males, the AUROC is higher for cases with imputed values compared to complete cases. However, calibration is worse for cases with imputed values compared to complete cases. Concerning the model for females, the AUROC is lower and calibration is worse for cases with imputed values compared to complete cases. In sum, the models are less suited for neighbourhoods consisting of fewer than 100 households.

**Table 5:** Results of the sensitivity analysis regarding imputations

		AUROC	E/O
Males	Complete cases	0.6540	0.9484
	Cases with imputations	0.6917	0.8694
Females	Complete cases	0.6755	0.9823
	Cases with imputations	0.6115	1.0675

*AUROC *area under the receiver operating characteristic, *E/O *expected-observed ratio

## Discussion

In this study, we developed and temporally evaluated sex-specific models to predict 4-year ASCVD risk based on age, as well as neighbourhoods’ socio-economic status and particulate matter 2.5 concentrations. We compared two model types, subdistribution and cause-specific hazard models, and eight model structures of varying complexity. The final model for males was a subdistribution hazard model including age, socio-economic status, and particulate matter as restricted cubic splines. The AUROC based on the evaluation data set equalled 0.6548. The overall expected-observed ratio equalled 0.9466. Calibration was deemed acceptable. The final model for females was a subdistribution hazard model including age, socio-economic status, and particulate matter as linear terms. The AUROC based on the evaluation data set equalled 0.6744. The overall expected-observed ratio equalled 0.9838. Calibration was deemed acceptable.

When comparing the subdistribution and cause-specific model types, we found only minor differences in performance. This is in line with a previous study by Wolbers et al. [51] These authors compared a non-competing risk Cox model, a subdistribution hazard model, and a cause-specific hazard model to predict 10-year risk of coronary heart disease in 55 to 90-year-old females living in Rotterdam. While the non-competing risk Cox model overestimated the risk, the subdistribution and cause-specific hazard models performed similarly.

With respect to the different model structures, we note age is the dominant predictor affecting model performance. Modelling age using a restricted cubic spline did not improve discrimination for both males and females, however it did improve calibration slightly for males only. Extending the model including age as a linear term with socio-economic status as a linear term improved discrimination modestly for both males and females, and calibration for females only. Likewise for particulate matter, this improved discrimination slightly for both males and females, and calibration for males only. Comparing the model including all predictors as linear terms to the model including all predictors as restricted cubic splines, discrimination and calibration was improved slightly for males only.

Notably, the final models’ discrimination seems on the low end compared to cardiovascular prediction models for similar populations that include clinical information, such as systolic blood pressure and lipid levels [19, 52]. The C-index of the SCORE2 models externally validated on a Dutch cohort equalled 0.721 [19]. The median C-index in external validation of the previous SCORE models and similar cardiovascular prediction models used in clinical practice ranged from 0.66 to 0.79 in a systematic review by Damen et al. [52]. However, our models are intended to be used before clinical evaluation, i.e. blood pressure measurements, blood tests, or other tests have not been performed. The only known information needed is the patient’s age, sex, and postal code. If these models are robust in future studies, these may be used in public health interventions to screen people without known ASCVD risk factors and refer those at high risk for further clinical evaluation. In addition, future research may extend the current models with clinical predictors. Subsequently, a stepwise implementation of both may be investigated.

Our study is limited by excluding people without a known address, and by failing to censor people moving out of the region. In addition, our study is limited by the restricted length of follow-up imposed by the availability of the data. Moreover, no suitable data regarding arsenic, cadmium, and lead were found. Future research may address these limitations, e.g. by censoring people at the date of moving, by updating the models using data with a longer follow-up period, or by collecting missing data. In addition, future research may investigate a minimum age per neighbourhood to invite patients for screening. In conclusion, we developed and temporally evaluated a model for males and females to predict 4-year ASCVD risk in South Limburg, the Netherlands, based on age, socio-economic status, and particulate matter 2.5 concentrations. The performance was deemed sufficient to explore its potential further in future research, with the long-term objective to aid screening people without known ASCVD risk factors.

## Supplementary Information

Supplementary Material 1: TRIPOD+AI checklists.

## Data Availability

Data from Statistics Netherlands, the Dutch Hospital Data foundation, and the Dutch National Institute for Public Health and the Environment were combined. Open data are possible to access via the references. Part of the data is not publicly available.

## Abbreviations

ASCVD: Atherosclerotic cardiovascular disease
AUROC: Area under the receiver operating characteristic
CSC: Cause-specific hazard model
E/O: Expected-observed ratio
ESC: European Society of Cardiology
ICD-10: International Statistical Classification of Diseases and Related Health Problems, 10th Revision
PM: Particulate matter
rcs: Restricted cubic spline
SCORE: Systematic Coronary Risk Evaluation
SD: Standard deviation
SDH: Subdistribution hazard model
SES: Socio-economic status
TRIPOD+AI: Transparent Reporting of a Multivariable Prediction Model for Individual Prognosis or Diagnosis + Artificial Intelligence
